# Toll-Like Receptors/TNF-*α* Pathway Crosstalk and Impact on Different Sites of Recurrent Myocardial Infarction in Elderly Patients

**DOI:** 10.1155/2022/1280350

**Published:** 2022-04-05

**Authors:** Xia Li, Dianxuan Guo, Ying Chen, Youdong Hu

**Affiliations:** Department of Geriatrics, The Affiliated Huaian Hospital of Xuzhou Medical University, Huaian 223002, China

## Abstract

**Background:**

Recurrent myocardial infarction is associated with increased mortality. Risk and predictive factors of recurrent myocardial infarction in elderly patients after coronary stenting are not well known. This research sought to investigate the effects of proinflammatory cytokines and toll-like receptor on recurrent myocardial infarction after coronary stenting in elderly patients.

**Methods:**

We measured the levels of toll-like receptor 2 (TLR2), toll-like receptor 3 (TLR3), toll-like receptor 4 (TLR4), tumor necrosis factor-*α* (TNF-*α*), soluble tumor necrosis factor-*α* receptor-1 (sTNFR-1), soluble tumor necrosis factor-*α* receptor-2 (sTNFR-2), endothelial progenitor cells (EPCs), and vascular endothelial growth factor (VEGF) in elderly patients with recurrent myocardial infarction and assessed the changes of proinflammatory cytokines and toll-like receptors in elderly patients with recurrent myocardial infarction after coronary stenting.

**Results:**

Levels of TLR2, TLR3, TLR4, TNF-*α*, sTNFR-1, and sTNFR-2 were remarkably increased (*P* < 0.001), and EPCs and VEGF were remarkably lowered (*P* < 0.001) in the elderly patients with recurrent myocardial infarction after coronary stent implantation. Increased expressions of proinflammatory cytokines and toll-like receptors induced recurrent myocardial infarction after coronary stenting. Elevated expressions of proinflammatory cytokines and toll-like receptors may be used to identify elderly patients who have an increased risk of developing recurrent myocardial infarction after coronary stenting.

**Conclusion:**

The increase levels of proinflammatory cytokines and toll-like receptors were associated with recurrent myocardial infarction after coronary stenting. Increased expressions of proinflammatory cytokines and toll-like receptors may be clinically useful biomarkers for predicting recurrent myocardial infarction in the elderly patients after coronary stent implantation.

## 1. Introduction

Compared to patients without recurrent myocardial infarction, patients who suffered from recurrent myocardial infarction were mainly old patients, more commonly in women, and rate of recurrent myocardial infarction was 4.1% among patients over 65 years of age in China. Coronary stent implantation triggered coronary endothelial inflammatory response, oxidative stress, injury, denudation, and dysfunction [1]. The local and systemic inflammations were documented in the patients with myocardial infarction, which included the increased expression of circulating proinflammatory cytokines. The systemic inflammation aggravated coronary atherosclerosis, and biomarkers for systemic inflammatory response were the predictors of recurrent myocardial infarction in patients with myocardial infarction [2]. The myocardial infarction was involved in the acute inflammatory response that led to coronary plaque disruption. The site of coronary plaque was infiltrated by inflammatory cells, and the persistent inflammatory response played a key role in destabilizing the coronary plaque and fibrous tissue cap [3].

Toll-like receptor 2 (TLR2), toll-like receptor 3 (TLR3), and toll-like receptor 4 (TLR4) through the adaptor molecule myeloid differentiation factor 88 were associated with noninfectious inflammatory response and initiated multiple intracellular signaling pathways contributing to the induction of proinflammatory mediators and the activation of transcription factors involved in inflammatory response [4–6]. The levels of TLR3 and TLR4 also were increased significantly in the patients with coronary in-stent restenosis [7] and were the potential noninvasive biomarkers for the clinical prognosis of coronary in-stent restenosis [8].

Tumor necrosis factor-*α* (TNF-*α*) was mainly produced by monocytes and macrophages. TNF-*α* was a pleiotropic inflammatory cytokine that promoted the proinflammatory response in coronary atherosclerosis and was also risk factor for coronary artery disease, and circulating TNF-*α* levels were related to increased risks of stable angina pectoris, unstable angina, and recurrent myocardial infarction [9–11]. TNF-*α* elicited inflammatory response via two receptors termed soluble TNF-*α* receptor-1 and 2 (sTNFR-1 and sTNFR-2). The sTNFR-1 and sTNFR-2 had key roles in cellular responses to stress and proinflammatory cytokine cascades, and sTNFR-1 and sTNFR-2 signaling pathways were important for developing cardiovascular disease [12]. The TNF-*α*/TNFR-1/TNFR-2 signaling pathways induced proinflammatory response [13]. TNF-*α* was considered to have proinflammatory effects via binding to its TNFR-1 and TNFR-2 receptors [14], and the expressions of TNFR1 and TNFR-2 were associated with higher risk of myocardial infarctions [15].

Endothelial progenitor cells (EPCs) were identified as a circulating cell population in the peripheral blood, and EPCs were incorporated into sites of neovascularization and promoted angiogenesis and participated in vascular repair after ischemic injuries [16, 17]. Inflammatory response was associated with lower EPC counts which lead to increased heart disease risks [18], and EPCs can ameliorate inflammatory response. The circulating EPCs as cell treatment for patients suffering from cardiovascular diseases [19] had the important prognostic implications in cardiac diseases and played a major role in repairing endothelial injuries [20]. Vascular endothelial growth factor (VEGF) acted via specific tyrosine kinase receptors and was a mitogen for vascular endothelial cells and stimulated angiogenesis and myocardial perfusion. VEGF promoted cell proliferation and migration and improved vascular scaffold endothelization [17, 21, 22]. VEGF exerted anti-inflammatory activity [23], and continuous release of VEGF to the lesion site of culprit vessels was proved be able to decrease inflammatory response. VEGF played an important role in anti-inflammatory response [24] and inhibited the proinflammatory reaction [25]. This research sought to investigate the clinical significance of level changes of TLR2, TLR3, TLR4, TNF-*α*, sTNFR-1, sTNFR-2, EPCs, and VEGF in elderly patients with recurrent myocardial infarction after coronary stent implantation.

## 2. Patients and Methods

### 2.1. Study Population

From 19 January 2014 to 11 March 2019, the present study included all consecutive patients with old recurrent myocardial infarction (RMI) after coronary artery implantation. The recurrent old RMI was defined as later than 14 days from onset in elderly patients after successful coronary stenting for myocardial infarction. The inclusion criteria established in this study were (1) patients aged 65 to 88 years old and (2) the patients with RMI after coronary stenting. The study was approved by the Affiliated Huaian Hospital of Xuzhou Medical University according to the relevant laws of China, and all patients gave their written informed consent before participation in this study according to the ethical guidelines of the Declaration of Helsinki. The following criteria were excluded from our study: (1) the uses of anti-inflammatory-immune drugs, (2) immune-modulating treatments, (3) clinical presentation with acute coronary syndromes, (4) recent coronary artery occlusions and myocardial infarction, (5) acute and chronic heart failure, (6) different malignant tumors, (7) allergic reactions to iodinated contrast media, (8) acute and chronic cerebral apoplexy, (9) severe systolic and diastolic hypertension, (10) cardiopulmonary-cerebral resuscitation, (11) severe acute and chronic liver diseases, (12) severe endocrine diseases, (13) acute and chronic hemodialysis, (14) acute and chronic venous vascular occlusions, and (15) acute and chronic inflammatory and immune diseases.

### 2.2. Research Protocol

**Figure 1:**
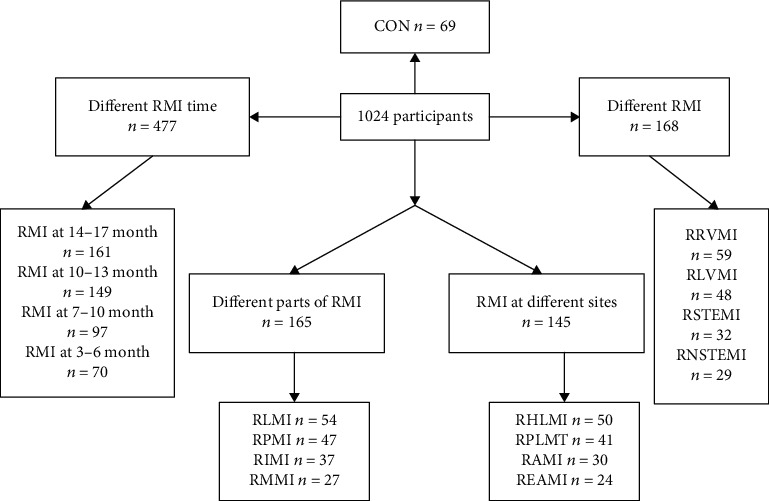
Flowchart of selection of eligible study subjects. RMI: recurrent myocardial infarction; RRVMI: recurrent right ventricular myocardial infarction; RLVMI: recurrent left ventricular myocardial infarction; RSTEMI: recurrent ST-elevation myocardial infarction; RNSTEMI: recurrent non-ST elevation myocardial infarction; RLMI: recurrent lateral myocardial infarction; RPMI: recurrent posterior myocardial infarction; RIMI: recurrent inferior myocardial infarction; RMMI: recurrent multiterritory myocardial infarction; RHLMI: recurrent high lateral myocardial infarction; RPLMI: recurrent postero-lateral myocardial infarction; RAMI: recurrent anterior myocardial infarction; REAMI: recurrent extensive anterior myocardial infarction.

The patients without RMI after coronary artery implantation were included in control group (*n* = 69). We evaluated the effects of TLR2, TLR3, TLR4, TNF-*α*, sTNFR-1, sTNFR-2, EPCs, and VEGF on different RMI time, different RMI, different parts of RMI, and different sites of RMI. According to RMI time, the patients were divided into RMI at 14-17 month group (*n* = 161), RMI at 10-13 month group (*n* = 149), RMI at 7-10 month group (*n* = 97), and RMI at 3-6 month group (*n* = 70). The patients were divided into recurrent right ventricular myocardial infarction (RRVMI) group (*n* = 59), recurrent left ventricular myocardial infarction (RLVMI) group (*n* = 48), recurrent ST-elevation myocardial infarction (RSTEMI) group (*n* = 32), and recurrent non-ST elevation myocardial infarction (RNSTEMI) group (*n* = 29) according to different RMI. According to different parts of RMI, the patients were divided into recurrent lateral myocardial infarction (RLMI) group (*n* = 54), recurrent posterior myocardial infarction (RPMI) group (*n* = 47), recurrent inferior myocardial infarction (RIMI) group (*n* = 37), and recurrent multiterritory myocardial infarction (RMMI) group (*n* = 27). The patients were further divided into recurrent high lateral myocardial infarction (RHLMI) group (*n* = 50), recurrent postero-lateral myocardial infarction (RPLMI) group (*n* = 41), recurrent anterior myocardial infarction (RAMI) group (*n* = 30), and recurrent extensive anterior myocardial infarction (REAMI) group (*n* = 24) according to RMI at different sites (Figure 1).

### 2.3. RMI Detection Using Coronary Angiography, Echocardiography, and Electrocardiography

RMI was detected by ambulatory electrocardiographic monitoring, the color Doppler echocardiograms were performed by two cardiac sonographers according to the guidelines of the American Society of Echocardiography Committee, and electrocardiographic examinations were performed by two cardiologists.

The patients underwent diagnostic coronary angiographies using the coronary angiography imaging system (QAngio XA MEDIS, Medical Imaging System BV, Leiden, The Netherlands). Diagnostic images of the coronary arteries were acquired from multiple view projections, avoiding overlap side branches and lesion foreshortening of relevant artery lesions. The coronary angiographies were diagnosed by two experienced clinical cardiologists. The significant coronary stenosis was defined as a stenosis ≥ 50% in at least one of three major coronary arteries. The extent of coronary stenosis is 50-100% (complete obstruction). Thrombolysis in myocardial ischemia (TIMI) flow grade was used to evaluate myocardial ischemia degree according to grade 0 defined as no flow, grade 1 defined as penetration without perfusion, grade 2 defined as partial perfusion, and grade 3 defined as complete perfusion of flow. Coronary complete occlusions were defined as completely occluded coronary arteries (100% stenosis) without residual anterograde coronary blood flow (myocardial infarction zero flow).

The successful crossing approaches to the lesions were antegrade wire escalation in 49.5%, retrograde wire escalation in 24.3%, reversal dissection reentry techniques in 11.4%, and reversal dissection reentry techniques in 14.8%. The procedural successes were defined as the successful guidewire and balloon crossing a completely coronary artery occluded lesions resulting in the successful dilation of the coronary occluded arteries and restoration of antegrade coronary flow (thrombolysis in myocardial infarction flow grade 3) with <50% residual lumen stenosis on final coronary angiographies. After the lesion was successfully crossed and coronary stents were implanted, the patients were enrolled in this research. A detailed description of indication and procedures of stent implantations is available in Supplemental Material Tables 1 and 2.

### 2.4. The Evaluation of TLR2, TLR3, and TLR4

Peripheral blood mononuclear cells were isolated from blood samples obtained from patients with RMI, and the expression of the TLR2, TLR3, and TLR4 on monocytes was measured using flow cytometry. In order to measure the levels of TLR2, TLR3, and TLR4 in the samples of patients with RMI, the venous blood samples were drawn at 8:00 AM and at 9:00 AM in the morning after an overnight fast (12 h), and serum samples for the measures of TLR2, TLR3, and TLR4 were centrifuged for 25 min at 3000×g. The levels of TLR2, TLR3, and TLR4 were expressed as mean fluorescence intensity (MFI) [26, 27].

### 2.5. The Evaluation of TNF-*α*, sTNFR-1, and sTNFR-2

The peripheral venous blood was collected at 8:00 AM and at 9:00 AM in the morning after an overnight fast (12 h) from all patients with RMI, and the serum samples were frozen and stored at -80°C until examination. According to manufacturer's specifications, plasma TNF*-α* levels were measured by ELISA using purified and biotinylated antihuman TNF*-α* monoclonal antibodies and recombinant human TNF*-α* (BD Biosciences Phar Mingen, San Diego, CA, USA). All samples were processed by ELISA plate reader (Bio-Rad, CA, USA) [28]. Serum was separated from blood samples and frozen at -80°C, and the levels of sTNFR-1 and sTNFR-2 were measured by ELISA kits (R&D Systems, Minneapolis, MN, USA) according to the manufacturer's specifications. The concentration of TNF-*α*, sTNFR-1, and sTNFR-2 were expressed as ng/L [28].

### 2.6. The Evaluation of EPC and VEGF

As an example, by using the source of information, we were able to analyze the levels of EPC and VEGF from the blood samples of healthy subjects and RMI patients and define the circulating biomarkers of EPC and VEGF for RMI, allowing risk prediction of RMI with accuracy in elderly patients. The flow cytometry for the evaluation of circulating EPCs from the patients with RMI was performed as previous reported work. Briefly, 200 *μ*l peripheral blood was immunostained for 20 to 30 min at room temperature using manufacturer-recommended concentrations with the antihuman CD34 monoclonal antibody (Becton Dickinson, R&D Systems, Minneapolis, MN, USA) and antihuman KDR (Sigma, Chemical Company, St. Louis, MO, USA), followed by the phycoerythrin-conjugated secondary antibody. The isotype-identical antibody controls (Becton Dickinson, R&D Minneapolis, MN, USA) and fluorescence-minus-one (FMO) controls were used to identify EPCs. The suspension was then incubated with FACS lysing solution (Becton Dickinson) according to manufacturer instructions for 10 to 15 min. After the incubation period, the cells were lysed and washed in phosphate-buffered saline and fixed with 4% paraformaldehyde solution before analysis of at least 70000 events. The numbers of circulating EPCs were counted by the flow cytometry and expressed as a percentage by independent blinded investigators [29]. The samples of peripheral venous plasma from control subjects and patients with RMI were collected into ice-cooled tubes containing an anticoagulant solution at 8:00 AM and at 9:00 AM in the morning after an overnight fast. Platelet-free plasma samples were prepared by a centrifugation method. The supernatants were frozen and stored at -80°C until assayed. Circulating VEGF plasma levels were measured in duplicate determinations using human VEGF ELISA kits (R&D, Systems, Minneapolis, Minnesota, USA). All plasma samples indicated high levels of VEGF ranging from 16 to 101 ng/L [30].

### 2.7. Data Analysis

The data analyses of the study results were expressed as the mean values ± standard deviations (mean ± SD). The paired Student's *t*-tests were used to determine each pair of the matching observed clinical data. The one-way analysis of variance was employed to evaluate the variance among the means. Multivariate regression analysis was used to identify the predictors of RMI after coronary stenting in patients. The statistically significant differences were considered to be present when significant differences (*P* < 0.05) were computed. All statistical analyses were carried out with the SPSS statistical package software for windows version 20.0 (SPSS, An IBM Corporation, Armonk, NY, USA) for the patients with RMI.

## 3. Results

### 3.1. Baseline Characteristics of the Elderly Patients with RMI

We enrolled 546 eligible patients aged 65 to 88 years old, including 278 males and 268 females. All patients completed the 17-month follow-up visit and were included in the analysis. The baseline patient characteristics were very similar within each study group of the elderly patients with RMI (Table 1). The patients in every group were well matched without statistically significant differences in age, gender, coronary heart disease, stable angina pectoris, unstable angina pectoris, previous myocardial infarction, family history of myocardial infarction, diabetes mellitus, hypertension, alcohol consumption, body weight, body height, and the duration of diseases.

### 3.2. Levels of TLR2, TLR3, TLR4, TNF-*α*, sTNFR-1, sTNFR-2, EPC, and VEGF in the Elderly Patients with Different RMI Time after Coronary Artery Implantation

The expression levels of TLR2, TLR3, TLR4, TNF-*α*, sTNFR-1, and sTNFR-2 were related to different RMI time. The levels of TLR2, TLR3, TLR4, TNF-*α*, sTNFR-1, and sTNFR-2 were increased significantly in RMI at 10-13 month group when compared with RMI at 14-17 month and CON groups, respectively (*P* < 0.001), and were further increased significantly in RMI at 3-6 month group compared to RMI at 7-10 month and RMI at 10-13 month groups, respectively (*P* < 0.001). Levels of EPC and VEGF were correlated with different RMI time, and levels of EPC and VEGF were independent predictors of different RMI time. Levels of EPC and VEGF were significantly decreased in RMI at 10-13 month group when compared with RMI at 14-17 month and CON groups respectively (*P* < 0.001) and were further decreased significantly in RMI at 3-6 month group compared to RMI at 7-10 month and RMI at 10-13 month groups respectively (*P* < 0.001).

These findings suggested that increased levels of TLR2, TLR3, TLR4, TNF-*α*, sTNFR-1, and sTNFR-2 and decreased levels of EPC and VEGF were associated with RMI time, and proinflammatory mediators and toll-like receptors were involved in the pathogenesis of RMI after coronary artery implantation in the elderly patients (Table 2).

### 3.3. Expressions of Proinflammatory Mediators and Toll-Like Receptors and Different RMI in the Elderly Patients after Coronary Artery Implantation

The patients underwent successful coronary stenting defined as residual stenosis < 50%, restoration of coronary flow (TIMI grade 3 flow), and no in-hospital major adverse cardiovascular events after coronary stenting. Implantations were successful in all elderly patients with residual stenosis < 50% and the restoration of TIMI grade 3 coronary flow, as assessed by coronary angiographies. There were no patients with no-reflow, microvascular obstruction cardiogenic shock, slow flow, perforations, coronary spasm, distal embolization, and abrupt closure events. No adverse clinical events were observed in any patient. The levels of TLR2, TLR3, TLR4, TNF-*α*, sTNFR-1, and sTNFR-2 were elevated in patients with different RMI compared with patients without RMI. Circulating levels of TLR2, TLR3, TLR4, TNF-*α*, sTNFR-1, and sTNFR-2 were obviously higher in RNSTEMI group than those in the other four different groups. The levels of TLR2, TLR3, TLR4, TNF-*α*, sTNFR-1, and sTNFR-2 were increased significantly in RLVMI group compared to RRVMI and CON groups, respectively (*P* < 0.001), and were further increased significantly in RNSTEMI group compared to RSTEMI and RLVMI groups respectively (*P* < 0.001). The decreased levels of EPC and VEGF were related to increased levels of TLR2, TLR3, TLR4, TNF-*α*, sTNFR-1, and sTNFR-2 in patients with different RMI. Levels of EPC and VEGF were significantly decreased in RLVMI group when compared with RRVMI and CON groups, respectively (*P* < 0.001), and were further decreased significantly in RNSTEMI group compared to RSTEMI and RLVMI groups, respectively (*P* < 0.001). These data supported the notion of possible involvement of proinflammatory mediators and toll-like receptors in different RMI after coronary artery implantation in the elderly patients (Table 3).

### 3.4. Expressions of TLR2, TLR3, TLR4, TNF-*α*, sTNFR-1, sTNFR-2, EPC, and VEGF and Different Parts of RMI in the Elderly Patients after Coronary Artery Implantation

The results demonstrated that the increased levels of circulating TLR2, TLR3, TLR4, TNF-*α*, sTNFR-1, and sTNFR-2 seemed to be related to low EPC and VEGF production and could well be responsible for the low EPC and VEGF levels in the elderly patients after coronary artery implantation. Levels of TLR2, TLR3, TLR4, TNF-*α*, sTNFR-1, and sTNFR-2 were increased significantly in RPMI group compared to RLMI and CON groups, respectively (*P* < 0.001), and were further increased significantly in RMMI group compared to RIMI and RPMI groups, respectively (*P* < 0.001). Levels of EPC and VEGF were significantly decreased in RPMI group when compared with RLMI and CON groups, respectively (*P* < 0.001), and were further decreased significantly in RMMI group compared to RIMI and RPMI groups, respectively (*P* < 0.001). The elderly patients with high levels of TLR2, TLR3, TLR4, TNF-*α*, sTNFR-1, and sTNFR-2 and decreased levels of EPC and VEGF had a significantly higher risk of developing different parts of RMI after coronary artery implantation (Table 4).

### 3.5. Expressions of Proinflammatory Cytokines and Toll-Like Receptors and RMI at Different Sites in the Elderly Patients after Coronary Stenting

We found that the concentrations of TLR2, TLR3, and TLR4 along with expression of TNF-*α*, sTNFR-1, and sTNFR-2 were each significantly higher in REAMI group compared with the other 4 groups. This was in contrast to decreased levels of EPC and VEGF in REAMI group as compared with the other 4 groups. The levels of TLR2, TLR3, TLR4, TNF-*α*, sTNFR-1, and sTNFR-2 were increased significantly in RPLMI group compared to RHLMI and CON groups, respectively (*P* < 0.001), and were further increased significantly in REAMI group compared to RAMI and RPLMI groups (*P* < 0.001). Levels of EPC and VEGF were significantly decreased in RPMI group when compared with RHLMI and CON groups, respectively (*P* < 0.001), and were further decreased significantly in REAMI group compared to RAMI and RPLMI groups, respectively (*P* < 0.001). The expressions of proinflammatory mediators and toll-like receptors seen in the elderly patients after coronary stenting were associated with RMI at different sites (Table 5).

### 3.6. TLR2, TLR3, TLR4, TNF-*α*, sTNFR-1, sTNFR-2, EPCs, and VEGF as Independent Indicators of RMI after Coronary Stenting

By multivariate regression analysis, TLR2, TLR3, TLR4, TNF-*α*, sTNFR-1, sTNFR-2, EPCs, and VEGF were found to be independent indicators of RMI after adjustment for age, gender, family history of myocardial infarction (MI), alcohol consumption, smoking, and diseases (stable angina pectoris, unstable angina pectoris, hypertension, and diabetes mellitus) in all patients. A *P* value of less than 0.05 was considered statistically significant (Table 6).

## 4. Discussion

Expressions of TLR2, TLR3, and TLR4 were demonstrated in atherosclerotic lesions and led to the development of the atherosclerotic lesions [31, 32], atherosclerotic arterial occlusion, and acute myocardial infarction [31, 32]. The high levels of TNF-*α* were closely related to the formation of atherosclerotic lesions and coronary heart disease [33]. TNF-*α* had the significant pro-inflammatory and oxidative stress effects on human coronary artery endothelial cells and promoted coronary artery endothelial dysfunction [34]. Inflammatory response in coronary restenosis after coronary stent implantation was related to high expression of TNF-*α* [35]. TNFR-1 and TNFR-2 played the key roles in inflammatory response, and the significant increase in the expressions of TNFR-1 and TNFR-2 was found in the arterial intimal thickening after coronary stenting. TNFR-1 and TNFR-2 were involved in coronary restenosis after coronary stenting [35].

Our study showed that the increased levels of TLR2, TLR3, TLR4, TNF-*α*, sTNFR-1, and sTNFR-2 were associated with RMI, and it supported the notion of possible involvement of proinflammatory mediators and toll-like receptors in the pathogenesis of RMI after coronary artery implantation in the elderly patients. Many previous reports showed that expressions of TLR2, TLR3, and TLR4 as proatherogenic receptors were related to the extent and severity of coronary heart disease and led to the development of the atherosclerotic lesions, atherosclerotic arterial occlusions, and acute myocardial infarction [31, 32]. Therefore, our results may indicate that TLR2, TLR3, and TLR4 significantly promoted RMI in association with significant inflammatory response in the elderly patients after coronary artery implantation. Many studies have been performed to assess the effects of proinflammatory cytokine TNF-*α*, TNFR-1, and TNFR-2 on formation of atherosclerotic lesions following coronary stenting. The high levels of TNF-*α* were closely related to the formation of atherosclerotic lesions and coronary heart disease [33]. TNF-*α* had the significant proinflammatory response and oxidative stress effects on human coronary artery and inflammatory response in coronary restenosis after coronary stent implantation was related to high expression of TNF-*α* [34, 35]. TNFR-1 and TNFR-2 were elevated in stented coronary arteries and were the key actors in the acute and the chronic inflammatory response following coronary stenting [35]. Therefore, based on our results and those from previous reports, TNF-*α*, sTNFR-1, and sTNFR-2 significantly promoted RMI related to significant inflammatory response in the elderly patients after coronary artery implantation. These findings showed that TLR2, TLR3, TLR4, TNF-*α*, sTNFR-1, and sTNFR-2 were the significantly higher risk factors of developing RMI and the potential noninvasive biomarkers for the clinical prognosis of RMI in elderly patients after coronary stenting.

It was revealed previously that the arterial endothelium dysfunction played a key role in atherosclerosis [36] and EPCs protected against the progression of atherosclerosis and the arterial intimal thickening [37]. The numbers of EPCs were decreased in atherosclerosis and myocardial infarction, and the mobilization of EPCs may be useful in clinical practice for coronary heart disease [38]. Consistent with previous findings, the levels of EPC were significantly decreased in the elderly patients with RMI after coronary artery implantation. It has been demonstrated that the roles of VEGF in vascular reformation included the inhibition of smooth muscle cell proliferation and local inflammatory response and the elevated content of EPCs [39, 40]. VEGF provided myocardial cell protection against myocardial damage. VEGF lnhibits oxidative stress in arterial endothelium and development and progression of atherosclerosis [41]. VEGF gene treatment enhanced the angiogenesis effects for salvaging ischemic myocardium and decreasing the myocardial infarct size [41]. The inhibition of coronary restenosis was the first target of VEGF gene treatment [40]. Our results are consistent with previous studies, and high levels of proinflammatory cytokines and toll-like receptors decreased the levels of VEGF and further accelerated development and progression of RMI after coronary artery implantation.

TLR2, TLR3, and TLR4 as biomarkers of proinflammatory response were increased significantly in patients with inflammatory bowel disease and breast cancer [42–44]. TLR4 was a pathophysiological link between inflammatory response and coronary atherosclerosis. The activation of TLR4 in vascular cells initiated the vascular inflammatory response and promoted coronary artery disease [45].

Proinflammatory cytokines (TNF-*α*, sTNFR-1, and sTNFR-2) were powerful independent predictors of chronic heart failure patients [46], and the inflammatory response was a core common factor in all stages of cardiac failure in patients. Expressions of sTNFR-1 and sTNFR-2 at the mRNA level were significantly increased in patients with cardiac failure [47].

The previous studies showed that clinical use of proinflammatory markers (TLR2, TLR2, TLR4, TNF-*α*, sTNFR-1, and sTNFR-2) provided a means of evaluating inflammatory diseases such as inflammatory bowel disease, breast cancer, coronary artery disease, chronic heart failure, and type 2 diabetes. The present study results demonstrated that the increase of the levels of proinflammatory mediators and toll-like receptors was associated with RMI after coronary stenting. Increased expressions of proinflammatory mediators and toll-like receptors may be clinically useful biomarkers for predicting RMI in the elderly patients after coronary stent implantation.

The myocardial infarction patients in the present study were treated with coronary stents, and coronary stent reocclusion was a severe medical event after coronary stenting. The coronary endothelial dysfunction and endothelial denudation were considered to be the risk of a major coronary event after coronary stent implantation [48, 49]. The coronary stent implantation promoted persistent endothelial inflammatory response, and the interaction of coronary endothelial inflammation and coronary endothelial dysfunction further led to coronary neointimal hyperplasia after coronary stent implantation [50, 51].

TLR2, TLR3, and TLR4 signaling pathways activated intracellular signaling cascades and elevated proinflammatory gene expression in human atherosclerotic plaques [52]. Proinflammatory cytokines (TNF-*α*, sTNFR1, and sTNFR2) were the important inflammatory mediators that were expressed during inflammatory responses [53]. The vascular endothelial dysfunction and injury were repaired by EPCs [54] and EPCs contributed to reendothelialisation. Inflammatory responses induce EPC dysfunction in humans, and the inflammatory stimulation had the adverse effect on circulating levels of EPCs [54]. VEGF mRNA expressions were predominantly observed in the vessel endothelial cells under the physiological conditions [55], and the anti-inflammatory effects of VEGF were related to downregulation of proinflammatory cytokines [55].

The present study demonstrated that the levels of proinflammatory mediators (TNF-*α*, sTNFR-1, and sTNFR-2) and toll-like receptors (TLR2, TLR3, and TLR4) were increased, and levels of anti-inflammatory mediators (EPCs and VEGF) were remarkably lowered significantly in the elderly patients with RMI after coronary artery implantation. The possible mechanisms for RMI after stent implantations suggested that the stent implantation induced persistent endothelial inflammatory response, coronary endothelial dysfunction, and endothelial denudation; inflammatory response may be directly associated with RMI; and proinflammatory mediators and toll-like receptors interplay were involved in the pathogenesis and progression of RMI after coronary artery implantation.

Our findings showed that the high levels of proinflammatory mediators and toll-like receptors markedly inhibited levels of EPC and VEGF. The decreased levels of EPC and VEGF in the elderly patients with RMI further accelerated development and progression of RMI. EPC and VEGF had good anti-inflammatory effects and promoted arterial healing. However, EPC and VEGF did not show marked anti-inflammatory response because TLR2, TLR3, TLR4, TNF-*α*, sTNFR-1, and sTNFR-2 inhibited expressions of EPC and VEGF in the present study.

## 5. Conclusion

The results of present study demonstrated that the levels of proinflammatory mediators (TNF-*α*, sTNFR-1, and sTNFR-2) and toll-like receptors (TLR2, TLR3, and TLR4) were increased significantly in the elderly patients with RMI after coronary artery implantation. Proinflammatory mediators and toll-like receptors interplay were involved in the pathogenesis and progression of RMI after coronary artery implantation. The levels of proinflammatory mediators and toll-like receptors might be considered as possible triggers for predicting, preventing, and treating RMI in the elderly patients after coronary artery implantation.

## Figures and Tables

**Table 1:** Baseline characteristics of elderly patients with RMI after coronary stent implantations.

	Without RMI *n* = 69	RMI at 14-17 months *n* = 161	RMI at 10-13 months *n* = 149	RMI at 7-10 months *n* = 97	RMI at 3-6 months *n* = 70
Age (years)	68.1 ± 3.0	68.3 ± 5.7	70.4 ± 5.6	75.8 ± 5.2	80.6 ± 7.4
Gender (male/female)	34/35	80/81	75/74	53/44	36/34
Coronary heart disease	0/0	83/78	78/71	47/50	33/37
Stable angina pectoris	0/0	44/41	42/32	24/26	15/17
Unstable angina pectoris	0/0	41/35	38/37	23/24	18/20
Remyocardial infarction	0/0	81/80	77/72	46/51	37/33
Previous myocardial infarction	0/0	81/80	77/72	46/51	37/33
Never smoker	0/0	32/30	24/22	16/15	11/10
Former smoker	0/0	29/26	26/20	14/18	14/12
Current smoker	0/0	20/24	27/30	16/18	12/11
Family history of MI	0/0	81/80	77/72	46/51	37/33
Drug-eluting stents	0/0	81/80	77/72	46/51	37/33
Diabetes mellitus	0/0	29/27	25/24	19/17	13/11
Hypertension	0/0	43/38	39/30	27/25	17/13
Alcohol consumption	0/0	34/32	35/32	20/18	15/13
Coronary angiography	0/0	81/80	77/72	46/51	37/33
Echocardiographic assessments	0/0	81/80	77/72	46/51	37/33
Severity of AS and RMI					
RMI with CTO	0/0	84/77	79/70	50/47	36/34
TIMI grade 0	0/0	79/82	76/73	45/52	40/30
RMI without angina	0/0	43/37	32/28	12/10	6/8
RMI with stable angina	0/0	25/20	24/26	19/17	14/12
RMI with unstable angina	0/0	19/17	21/18	20/19	17/13
Atrial arrhythmias	0/0	22/24	20/19	19/21	17/15
Ventricular arrhythmias	0/0	17/20	15/13	13/15	11/14
LVEF (%)	55 ± 5	56 ± 6	50 ± 5	49 ± 4	50 ± 7
PMIS	0/0	35/32	29/27	16/15	14/13

MI: myocardial infarction; AS: atherosclerosis; RMI: recurrent myocardial infarction; CTO: coronary total occlusion; TIMI: thrombolysis in myocardial ischemia flow; LVEF: left ventricular ejection fraction; PMIS: postmyocardial infarction syndrome.

**Table 2:** Biomarker levels and different RMI time in the elderly patients after coronary stent implantations.

	Without RMI *n* = 69	RMI at 14-17 months *n* = 161	RMI at 10-13 months *n* = 149	RMI at 7-10 months *n* = 97	RMI at 3-6 months *n* = 70
TLR2 (MFI)	4.7 ± 0.9	6.7 ± 1.3^∗^	8.9 ± 1.7^∗∗^	13.0 ± 2.6^∗∗∗^	16.2 ± 3.2^∗∗∗∗^
TLR3 (MFI)	2.7 ± 0.6	5.4 ± 1.1^∗^	7.9 ± 1.5^∗∗^	10.0 ± 2.0^∗∗∗^	14.5 ± 2.9^∗∗∗∗^
TLR4 (MFI)	2.3 ± 0.4	4.0 ± 0.8^∗^	7.6 ± 1.5^∗∗^	12.4 ± 2.1^∗∗∗^	16.8 ± 3.0^∗∗∗∗^
TNF-*α* (ng/L)	23.0 ± 4.6	44.1 ± 7.8^∗^	59.7 ± 10.4^∗∗^	81.3 ± 14, 2^∗∗∗^	110.6 ± 20.1^∗∗∗∗^
sTNFR-1 (ng/L)	118.3 ± 23.6	130.8 ± 26.0^∗^	171.4 ± 32.0^∗∗^	190.1 ± 37.1^∗∗∗^	259.8 ± 41.5^∗∗∗∗^
sTNFR-2 (ng/L)	139.1 ± 27.8	233.0 ± 36.6^∗^	291.0 ± 48.1^∗∗^	376.9 ± 72.3^∗∗∗^	469.0 ± 83.1^∗∗∗∗^
EPC (%)	7.3 ± 1.0	6.1 ± 1.2^∗^	4.7 ± 1.0^∗∗^	2.3 ± 0.4^∗∗∗^	1.2 ± 0.2^∗∗∗∗^
VEGF (ng/L)	13.0 ± 2.6	10.0 ± 2.0^∗^	7.3 ± 1.4^∗∗^	6.1 ± 1.2^∗∗∗^	2.0 ± 0.3^∗∗∗∗^
LVEF (%)	55 ± 5	45 ± 6	44 ± 8	41 ± 6	40 ± 7
SV (mL)	61 ± 7	63 ± 5	59 ± 7	54 ± 5	50 ± 7

^∗^ *P* < 0.001 (CON group/RMI at 14-17 month group). ^∗∗^*P* < 0.001 (RMI at 14-17 month group/RMI at 10-13 month group). ^∗∗∗^*P* < 0.001 (RMI at 10-13 month group/RMI at 7-10 month group). ^∗∗∗∗^*P* < 0.001 (RMI at 7-10 month group/RMI at 3-6 month group). LVEF: left ventricular ejection fraction; SV: stroke volume. There were no significant differences in LVEF and SV among the groups (*P* > 0.05).

**Table 3:** Biomarker levels and different RMI in the elderly patients after coronary stent implantations.

	Without RMI *n* = 69	RRVMI *n* = 59	RLVMI *n* = 48	RSTEMI *n* = 32	RNSTEMI *n* = 29
TLR2 (MFI)	4.7 ± 0.9	7.3 ± 1.4^∗^	9.7 ± 1.9^∗∗^	11.4 ± 2.2^∗∗∗^	14.7 ± 2.9^∗∗∗∗^
TLR3 (MFI)	2.7 ± 0.6	4.9 ± 1.1^∗^	6.7 ± 1.3^∗∗^	9.0 ± 1.8^∗∗∗^	13.7 ± 2.7^∗∗∗∗^
TLR4 (MFI)	2.3 ± 0.4	5.2 ± 1.0^∗^	8.0 ± 1.6^∗∗^	13.3 ± 2.5^∗∗∗^	17.0 ± 3.1^∗∗∗∗^
TNF-*α* (ng/L)	23.0 ± 4.6	31.0 ± 5.2^∗^	53.4 ± 9.6^∗∗^	76.5 ± 13.2^∗∗∗^	103.8 ± 20.7^∗∗∗∗^
sTNFR-1 (ng/L)	118.3 ± 23.6	140.2 ± 26.4^∗^	170.2 ± 32.1^∗∗^	197.0 ± 39.4^∗∗∗^	268.9 ± 43.7^∗∗∗∗^
sTNFR-2 (ng/L)	139.1 ± 27.8	143.1 ± 28.5^∗^	164.0 ± 31.6^∗∗^	190.0 ± 38.0^∗∗∗^	230.5 ± 45.1^∗∗∗∗^
EPC (%)	7.3 ± 1.0	5.4 ± 1.1^∗^	3.1 ± 0.5^∗∗^	2.6 ± 0.6^∗∗∗^	1.4 ± 0.2^∗∗∗∗^
VEGF (ng/L)	13.0 ± 2.6	11.0 ± 3.0^∗^	8.4 ± 3.6^∗∗^	5.6 ± 4.7^∗∗∗^	3.2 ± 7.5^∗∗∗∗^
LVEF (%)	55 ± 5	56 ± 6	50 ± 5	49 ± 4	50 ± 7
SV (mL)	61 ± 7	63 ± 8	60 ± 6	55 ± 7	56 ± 8

^∗^ *P* < 0.001 (CON group/RRVMI group). ^∗∗^*P* < 0.001 (RRVMI group/RLVMI group). ^∗∗∗^*P* < 0.001 (RLVMI group/RSTEMI group). ^∗∗∗∗^*P* < 0.001 (RSTEMI group/RNSTEMI group). LVEF: left ventricular ejection fraction; SV: stroke volume. There were no significant differences in LVEF and SV among the groups (*P* > 0.05).

**Table 4:** Biomarker levels and different parts of RMI in the elderly patients after coronary stent implantations.

	Without RMI *n* = 69	RLMI *n* = 54	RPMI *n* = 47	RIMI *n* = 37	RMMI *n* = 27
TLR2 (MFI)	4.7 ± 0.9	8.0 ± 1.5^∗^	11.0 ± 2.1^∗∗^	14.3 ± 2.8^∗∗∗^	17.5 ± 3.4^∗∗∗∗^
TLR3 (MFI)	2.7 ± 0.6	6.3 ± 1.2^∗^	9.5 ± 1.9^∗∗^	12.6 ± 2.4^∗∗∗^	15.9 ± 3.5^∗∗∗∗^
TLR4 (MFI)	2.3 ± 0.4	7.1 ± 1.4^∗^	10.3 ± 2.0^∗∗^	12.1 ± 2.4^∗∗∗^	16.8 ± 3.1^∗∗∗∗^
TNF-*α* (ng/L)	23.0 ± 4.6	41.3 ± 7.2^∗^	67.2 ± 10.4^∗∗^	85.3 ± 16.0^∗∗∗^	125.6 ± 24.0^∗∗∗∗^
sTNFR-1 (ng/L)	118.3 ± 23.6	151.4 ± 30.2^∗^	178.2 ± 35.6^∗∗^	201.5 ± 40.1^∗∗∗^	280.8 ± 54.2^∗∗∗∗^
sTNFR-2 (ng/L)	139.1 ± 27.8	152.0 ± 30.4^∗^	170.0 ± 34.0^∗∗^	189.8 ± 37.2^∗∗∗^	220.8 ± 42.1^∗∗∗∗^
EPC (%)	7.3 ± 1.0	6.7 ± 1.3^∗^	5.5 ± 1.1^∗∗^	4.6 ± 0.9^∗∗∗^	3.0 ± 0.4^∗∗∗∗^
VEGF (ng/L)	13.0 ± 2.6	9.9 ± 2.0^∗^	7.0 ± 1.4^∗∗^	4.3 ± 0.8^∗∗∗^	2.1 ± 0.3^∗∗∗∗^
LVEF (%)	55 ± 5	61 ± 7	54 ± 4	59 ± 8	58 ± 9
SV (mL)	61 ± 7	52 ± 5	48 ± 8	50 ± 6	49 ± 8

^∗^ *P* < 0.001 (CON group/RLMI group). ^∗∗^*P* < 0.001 (RLMI group/RPMI group). ^∗∗∗^*P* < 0.001 (RPMI group/RIMI group). ^∗∗∗∗^*P* < 0.001 (RIMI group/RMMI group). LVEF: left ventricular ejection fraction; SV: stroke volume. There were no significant differences in LVEF and SV among the groups (*P* > 0.05).

**Table 5:** Biomarker levels and RMI at different sites in the elderly patients after coronary stent implantations.

	Without RMI *n* = 69	RHLMI *n* = 50	RPLMI *n* = 41	RAMI *n* = 30	REAMI *n* = 24
TLR2 (MFI)	4.7 ± 0.9	7.0 ± 1.4^∗^	9.3 ± 1.8^∗∗^	13.0 ± 2.4^∗∗∗^	15.1 ± 3.0^∗∗∗∗^
TLR3 (MFI)	2.7 ± 0.6	5.1 ± 1.1^∗^	8.7 ± 1.7^∗∗^	10.0 ± 2.0^∗∗∗^	12.4 ± 2.4^∗∗∗∗^
TLR4 (MFI)	2.3 ± 0.4	3.9 ± 0.7^∗^	5.7 ± 1.1^∗∗^	9.1 ± 1.8^∗∗∗^	13.1 ± 2.6^∗∗∗∗^
TNF-*α* (ng/L)	23.0 ± 4.6	32.0 ± 5.3^∗^	59.8 ± 10.9^∗∗^	82.5 ± 15.4^∗∗∗^	107.2 ± 20.4^∗∗∗∗^
sTNFR-1 (ng/L)	118.3 ± 23.6	135.1 ± 26.0^∗^	169.6 ± 33.8^∗∗^	181.0 ± 35.2^∗∗∗^	200.4 ± 40.1^∗∗∗∗^
sTNFR-2 (ng/L)	139.1 ± 27.8	159.0 ± 31.5^∗^	180.7 ± 35.2^∗∗^	217.9 ± 42.5^∗∗∗^	241.3 ± 48.0^∗∗∗∗^
EPC (%)	7.3 ± 1.0	5.9 ± 1.2^∗^	3.7 ± 0.7^∗∗^	2.2 ± 0.4^∗∗∗^	1.6 ± 0.3^∗∗∗∗^
VEGF (ng/L)	13.0 ± 2.6	9.7 ± 1.8^∗^	6.9 ± 1.3^∗∗^	5.1 ± 1.0^∗∗∗^	3.9 ± 0.2^∗∗∗∗^
LVEF (%)	55 ± 5	47 ± 9	50 ± 5	48 ± 7	45 ± 6
SV (mL)	61 ± 7	63 ± 6	60 ± 8	47 ± 7	48 ± 5

^∗^ *P* < 0.001 (CON group/RHLMI group). ^∗∗^*P* < 0.001 (RHLMI group/RPLMI group). ^∗∗∗^*P* < 0.001 (RPLMI group/RAMI group). ^∗∗∗∗^*P* < 0.001 (RAMI group/REAMI group). LVEF: left ventricular ejection fraction; SV: stroke volume. There were no significant differences in LVEF and SV among the groups (*P* > 0.05).

**Table 6:** Multivariate regression analysis to evaluate the significance of variables for RMI after coronary stenting.

Variables	Odds ratio	95% CI	*P* value
Age	1.64	0.98-2.71	0.09
Gender	1.45	0.75-1.67	0.43
Stable angina pectoris	1.31	0.74-1.96	0.16
Unstable angina pectoris	1.14	0.56-1.68	0.42
Never smoker	1.34	0.85-1.84	0.18
Former smoker	0.51	0.12-1.23	0.06
Current smoker	0.21	0.71-1.30	0.60
Alcohol consumption	2.17	0.17-7.33	0.08
Hypertension	2.01	0.47-6.73	0.43
Diabetes mellitus	1.60	0.41-5.54	0.45
Family history of MI	1.83	0.27-2.63	0.80
TLR2	1.39	1.23-2.89	0.01
TLR3	4.21	2.32-7.24	0.03
TLR4	4.10	2.81-6.10	0.001
TNF-*α*	1.21	1.18-2.70	0.002
sTNFR-1	2.15	1.40-3.13	0.01
sTNFR-2	1.82	1.21-2.63	0.01
EPCs	3.37	2.80-7.21	0.02
VEGF	4.08	2.79-5.40	0.03

## Data Availability

The data used to support the findings of this study are available from the corresponding author upon request.

## Supplementary Materials

Supplementary Materials Supplemental Table 1: indication of direct stenting of coronary artery. Supplemental Table 2: procedural characteristics of coronary occlusions and PCI.
