# Supplementary material for: Toll-Like Receptors/TNF-α Pathway Crosstalk and Impact on Different Sites of Recurrent Myocardial Infarction in Elderly Patients
Source: Biomed Res Int. 2022 Apr 5;2022:1280350. doi: 10.1155/2022/1280350 (PMC9005286; doi:10.1155/2022/1280350)
Supplement: Supplementary Materials — Supplemental Table 1: indication of direct stenting of coronary artery. Supplemental Table 2: procedural characteristics of coronary occlusions and PCI. [file 1280350.f1.doc]

Supplemental Table 1. Indication of direct stenting of coronary artery.

|  | Right coronary occlusion  n = 313 | Left circumflex occlusion  n = 319 | Left anterior descending occlusion  n = 323 |
| --- | --- | --- | --- |
| Indication for coronary stents |  |  |  |
| Myocardial infarctions, % | 50 | 41 | 19 |
| Occlusion+stable angina, % | 33 | 34 | 32 |
| Occlusion+unstable angina, % | 17 | 25 | 49 |
| *P* values | 0.01 | 0.04 | 0.04 |

Supplemental Table 2. Procedural characteristics of coronary occlusions and PCI.

|  | Right coronary occlusion  n = 313 | Left circumflex occlusion  n = 319 | Left anterior descending occlusion  n = 323 |
| --- | --- | --- | --- |
| **Vascular Access in PCI** |  |  |  |
| Femoral artery, % | 63 | 47 | 40 |
| Radial artery, % | 25 | 33 | 33 |
| Femoral+radial artery, % | 12 | 20 | 27 |
| *P* values | 0.03 | 0.01 | 0.02 |
| **Target coronary arteries** |  |  |  |
| Distal coronary artery, % | 50 | 44 | 20 |
| Middle coronary artery, % | 34 | 34 | 34 |
| Proximal coronary artery, % | 16 | 22 | 46 |
| *P* values | 0.02 | 0.03 | 0.02 |
| **Different stent lengths** |  |  |  |
| 13-26 mm, % | 45 | 25 | 20 |
| 30-40mm, % | 34 | 33 | 34 |
| 46-56mm, % | 21 | 42 | 46 |
| *P* values | 0.03 | 0.02 | 0.02 |
| **Different stent implantations** |  |  |  |
| One short stent, % | 45 | 17 | 20 |
| One single long stent, % | 33 | 33 | 33 |
| Multiple overlapped stents, % | 22 | 50 | 47 |
| *P* values | 0.02 | 0.01 | 0.01 |
| **Procedure time of PCI** |  |  |  |
| 42 minutes, % | 53 | 10 | 19 |
| 104 minutes, % | 32 | 35 | 32 |
| 127 minutes, % | 15 | 55 | 49 |
| *P* values | 0.04 | 0.04 | 0.04 |

PCI, percutaneous coronary intervention.
